# Burden of Non-cardiac Patients on the Emergency Room of a Rural Cardiac Center in Sindh, Pakistan

**DOI:** 10.7759/cureus.3291

**Published:** 2018-09-12

**Authors:** Jawaid A Sial, Naveedullah Khan, Waheed Murad, Musa Karim

**Affiliations:** 1 Cardiology, National Institute of Cardiovascular Diseases (NICVD), Karachi, PAK; 2 Cardiology, Sheikh Khalifa Medical City, Abu Dhabi, ARE; 3 Research, National Institute of Cardiovascular Diseases (NICVD), Karachi, PAK

**Keywords:** chest pain, cardiac, non-cardiac, emergency, burden

## Abstract

Introduction

The number of cardiac patients increases on a daily basis, and emergency departments bear much of the burden of non-cardiac patients due to pathological fears of the aftermath of the disease. Therefore, this study aimed to determine the burden of non-cardiac patients on the emergency department of a cardiac center in a rural area of Sindh, Pakistan.

Methods

This cross-sectional study was conducted at the emergency department of Chandka Medical College Hospital in Larkana. Consecutive patients who presented with cardiac symptoms with no previous history of cardiac disease were included. After a brief history, physical examination, electrocardiogram, and a cardiac enzyme assessment, patients were categorized as cardiac or non-cardiac. Data were analyzed using IBM SPSS Statistics for Windows, Version 21.0. (IBM Corp., Armonk, NY, US) and p ≤0.05 was statistically significant.

Results

Of the 204 patients included, 112 (59.8%) were men, and the mean age was 47 ± 16 years. Most patients (n = 146; 71.6%) were diagnosed as non-cardiac. The non-cardiac diagnosis was significantly more common among patients without diabetes (n = 123, 77.4% vs. n = 23, 51.1%; p = 0.001), without chest pains (n = 93, 81.6% vs. n = 53, 58.9%; p< 0.001), and without shortness of breath (n = 107, 75.9% vs. n = 39, 61.9%; p = 0.041).

Conclusion

More than two-thirds of the patients were found to have a non-cardiac mechanism behind their symptoms. A major proportion of the emergency room's cardiology department is occupied by non-cardiac patients. Owing to its direct and indirect implication on an otherwise struggling health system, we suggest chest pain units should be developed to decrease the workload and provide better care to cardiac patients.

## Introduction

According to the Institute of Health Metrics and Evaluation profile of Pakistan, ischemic heart disease (IHD) and cerebrovascular disease were the leading causes of death in 2016, with a percentage increase of 23.0% and 13.4% from 2005 to 2016, respectively. IHD was also the leading cause of premature deaths, with a percentage increase of 21.4% from 2005 to 2016 [1]. With over 207 million inhabitants, Pakistan has endured a major portion of the global burden of deaths due to cardiovascular diseases (CVDs) [2]. Among other environmental and heritable factors that aggravate the prevalence and subsequent mortality due to CVD, overcrowding emergency departments in an already limited health care system in Pakistan is a serious problem for reliable health care [3-6].

Recent studies have declared the state of emergency care in Pakistan as sub-optimal, with deficiencies in both human resources as well as the availability of equipment and levels of supplies [4,7]. In such a resource-limited health care system, the degree of burden of non-cardiac patients on a cardiac emergency department is worrisome. Chest pain is the leading precipitant of cardiac emergency department visits all over the world [8-11]. Chest pain categorized as non-cardiac in origin accounts for around 45% to 77% of the patients presenting to emergency departments [10,12-18]. This substantially high frequency of non-cardiac chest pain can be attributed to the increasing awareness among the general population regarding the lethal manifestation and pathological fears of the aftermath of heart disease. This pathological fear is termed cardiophobia and is prevalent among nearly half of the patients with a non-cardiac chest pain [13,19-20].

The most commonly found underlying mechanisms of non-cardiac chest pain reported in past studies are gastroesophageal reflux disorder (GERD), esophageal hypersensitivity and dysmotility, musculoskeletal pain, major depressive disorder (MDD), and pericarditis [8,13,21].

Non-cardiac chest pain is not only a financial burden on the health care system but also results in the inappropriate use of health care facilities [10]. A study in the United States estimated the cost of the initial care of patients ultimately diagnosed with non-cardiac disease is around eight billion dollars [15]. In countries like Pakistan, where the health care provider-to-seeker ratio is already imbalanced, non-cardiac chest pain is a major concern, with both direct and indirect impacts on the health care system, in terms of health care cost and utilization. Therefore, the aim of this study was to determine the burden of non-cardiac patients on the emergency department of a cardiac center in the rural areas of Sindh, Pakistan.

## Materials and methods

This cross-sectional study was conducted at the emergency department of Chandka Medical College, Larkana, from January 15, 2015, to April 14, 2015. After receiving approval of the study design from our institutional ethical review committee, we conducted a review of the consecutive patients who presented to the emergency department with concerns of chest pain, shortness of breath (SOB), palpitation, or syncope. The importance and befits of the study were explained, and we obtained informed consent from all enrolled patients or their legal caretakers. Patients with a prior history of acute coronary syndrome (ACS) or congenital heart disease were excluded. We reviewed patient demographic details, such as age, gender, relevant medical history (e.g., family history of premature coronary artery diseases, personal history of hypertension, smoking, and diabetes mellitus), and presenting concerns such as chest pain, SOB, palpitation, and syncope. After a brief history and examination, electrocardiographic assessment, and cardiac enzyme test (where needed), patients were categorized as “cardiac” or “non-cardiac.” To avoid the observational bias, patient reports were assessed by three independent cardiologists with more than five years’ experience, each blinded to the diagnosis by the remaining two, and patients were classified as cardiac or non-cardiac cases based on an agreement of at least two of the cardiologists. The sample size for the study was calculated with an expected prevalence of non-cardiac symptoms as 55% [14], a 95% confidence level, and a margin of error of 7%. Based on these assumptions, the calculated sample size was 195 patients; we recruited 10 additional patients to ensure set precision in the event of information loss. Of the 205 patients enrolled, one patient was excluded from the analysis due to missing information on the required variables.

Data were collected on predesigned structural proforma. Collected data were entered and analyzed using IBM SPSS Statistics for Windows, Version 21.0. (IBM Corp., Armonk, NY, US). Categorical variables were expressed as frequency and percentage. Minimum, maximum, and mean ± standard deviation (SD) were calculated for continuous variables. The chi-square test was performed to assess the outcome by demographic and baseline characteristics. Multivariate logistic regression was applied to assess the effect of patients’ baseline characteristics on non-cardiac diagnosis. A two-sided p-value of ≤ 0.05 was taken as criteria for statistical significance.

## Results

Of the 204 included patients, 112 (59.8%) were men, and the mean age (± SD) of the patients was 47 ± 16 years with a minimum age of 14 years and a maximum age of 90 years. Most patients (n = 129, 63.2%) were 50 years or younger. More than half of the patients (n = 112, 54.9%) were hypertensive. Seventeen patients (8.3%) had a family history of premature coronary artery diseases (CAD). Ninety-nine patients (44.1%) presented to the emergency department with chest pain while SOB was the second most common presenting concern. Clinical history and presenting concerns are presented in Table 1.

**Table 1:** Clinical history and presenting concerns Abbreviations: SD, Standard Deviation; CAD, Coronary Artery Diseases

Baseline Characteristics	Frequency (%)
Gender	
Male	122 (59.8%)
Female	82 (40.2%)
Age [Mean ± SD]	47.33 ± 15.83 years
Up to 50 years	129 (63.2%)
More than 50 years	75 (36.8%)
Clinical History	
Family History CAD	17 (8.3%)
Hypertension	112 (54.9%)
Diabetes Mellitus	45 (22.1%)
Smoking	31 (15.2%)
Presenting Concern	
Chest Pain	90 (44.1%)
Shortness of Breath	63 (30.9%)
Palpitation	21 (10.3%)
Syncope	2 (1%)

One hundred forty-six patients (71.6%) had a non-cardiac mechanism behind their symptoms. Cardiac abnormalities were diagnosed in 58 patients (28.4%). Of these cardiac patients, 38 (65.5%) had ACS, 11 (19.0%) had valvular abnormalities, and nine (15.5%) had heart failure. Patients without diabetes were more likely to have a non-cardiac diagnosis (p = 0.001). Similarly, patients who presented without chest pain or SOB and with palpitation were more likely to have a non-cardiac diagnosis (p < 0.001, p = 0.041, and p = 0.043, respectively). The final diagnoses, according to baseline characteristics and presenting concerns, are presented in Table 2.

**Table 2:** Final diagnosis by baseline characteristics of the patients Abbreviations: CAD, Coronary Artery Diseases ** P-values are based on the chi-square test * Significant at the 5% level of significance

Baseline Characteristics	Base	Final Diagnosis		**p-value
Frequency (row %)	N	Cardiac	Non-Cardiac	
Gender				
Male	122	33 (27%)	89 (73%)	0.593
Female	82	25 (30.5%)	57 (69.5%)	
Age				
Up to 50 years	129	36 (27.9%)	93 (72.1%)	0.828
More than 50 years	75	22 (29.3%)	53 (70.7%)	
Family History CAD				
Yes	17	3 (17.6%)	14 (82.4%)	0.303
No	187	55 (29.4%)	132 (70.6%)	
Hypertension				
Yes	112	32 (28.6%)	80 (71.4%)	0.961
No	92	26 (28.3%)	66 (71.7%)	
Diabetes Mellitus				
Yes	45	22 (48.9%)	23 (51.1%)	0.001*
No	159	36 (22.6%)	123 (77.4%)	
Smoking				
Yes	31	10 (32.3%)	21 (67.7%)	0.608
No	173	48 (27.7%)	125 (72.3%)	
Chest Pain				
Yes	90	37 (41.1%)	53 (58.9%)	<0.001*
No	114	21 (18.4%)	93 (81.6%)	
Shortness of Breath				
Yes	63	24 (38.1%)	39 (61.9%)	0.041*
No	141	34 (24.1%)	107 (75.9%)	
Palpitation				
Yes	21	2 (9.5%)	19 (90.5%)	0.043*
No	183	56 (30.6%)	127 (69.4%)	
Syncope				
Yes	2	0 (0%)	2 (100%)	0.37
No	202	58 (28.7%)	144 (71.3%)	

On a multivariate logistic regression model with a non-cardiac diagnosis as a dependent variable (Hosmer and Lemeshow test p-value = 0.611), presenting without diabetes, chest pain, or SOB was significant (p = 0.005, <0.001, and p = 0.003, respectively; odds ratios of 2.88, 4.54, and 3.37, respectively) while palpitation at presentation was found to be insignificant (p= 0.113).

## Discussion

This study was conducted to determine the burden of non-cardiac patients on a cardiac emergency department. We found more than two-thirds (71.6%) of the patients who visited the cardiac emergency room with concerns of chest pain, SOB, palpitation, or syncope had a non-cardiac mechanism behind their symptoms. A cardiac symptom (e.g., chest pain) eventually diagnosed to be of non-cardiac origin is the pertinent problem every cardiac emergency room faces. The frequency of non-cardiac origin for symptoms in our study falls within the range of 45% to 77% reported in previous studies [10,12-18]. There is always uncertainty among physicians regarding discharging patients with no viable explanation for their symptoms. There is always the fear of missed diagnosis and overtreatment, which may result in life-threatening circumstances or side effects from unnecessary medication and higher health care costs [11]. In our study, the univariate and multivariate analyses revealed that the non-cardiac diagnosis among the patients who presented with cardiac symptoms was significantly higher among patients without diabetes (n = 123, 77.4%) compared to those with diabetes (n =23, 51.1%; p = 0.001). Patients’ presenting concerns have certain clues to indicate non-cardiac origins; for example, patients presenting without chest pain are 4.54 times more likely to have a non-cardiac origin of their symptoms with a frequency comparison of 93 (81.6%) vs. 53 (58.9%), p < 0.001. Similarly, patients presenting without SOB are 3.37 times more likely to be diagnosed as non-cardiac, with a frequency compassion of 107 (75.9%) vs. 39 (61.9%), p = 0.041.

We did not determine the cause of symptoms for the non-cardiac patients. However, various causes have been identified over the years for non-cardiac symptoms, especially non-cardiac chest pain. The most common causes are GERD, esophageal hypersensitivity and dysmotility, musculoskeletal pain, MDD, and pericarditis [8,13,21].

The high burden of non-cardiac patients on a resource-strained cardiac emergency room in a developing country like Pakistan [4,7] is a serious issue that warrants the attention of both health care professionals and governing bodies. It is important to focus on developing chest pain units for the rapid assessment of non-cardiac causes and patient risk assessments [22]. Various efficient, cost-effective, and easily adoptable risk stratification modalities have been proposed in the literature, such as the report by Backus et al. [11], who proposed a score composed of patient history, electrocardiogram, age, risk factors, and troponin levels (HEART), for accurate decision-making and triage. Soares-Filho et al. [14] reported a significant proportion of psychiatric illness among the patients with symptoms indicative of chest pain and recommended to apply the hospital anxiety and depression scale (HADS) method for the screening of such patients. Al-Ani et al. [13] found GERD, depression, and anxiety more common than CAD in patients presenting with acute chest pain. Similarly, Hadlandsmyth et al. [15] reported a significant association of interoceptive fear and anxiety with health care utilization among patients with chest pain.

Limitations

Our single-center study’s small sample size and geographic coverage limit the generalizability of our findings; for a thorough understanding of the non-cardiac burden on the cardiac emergency room, larger multicenter studies are needed.

## Conclusions

More than two-thirds of the patients were found to have a non-cardiac mechanism behind their symptoms. A major part of emergency room cardiology resources is occupied by non-cardiac patients. Owing to its direct and indirect implication on an otherwise struggling health system, we suggest chest pain units should be developed to decrease the workload and provide better care to cardiac patients.
